# Incidental Detection of Bilateral Node-Positive Papillary Thyroid Carcinoma During Initial Staging for Localized Testicular Seminoma: A Case Report

**DOI:** 10.7759/cureus.117467

**Published:** 2026-10-05

**Authors:** Roman Farnin, Halil Altindag, Antonia Hammer, Robert Lienenlüke, Johanna Engler, Lula Gebrehiwot, Julian Mittermeier, Andrea Sergio Merlo, Christian Vorländer

**Affiliations:** 1 Department of Endocrine Surgery, Bürgerhospital Frankfurt am Main, Frankfurt am Main, DEU

**Keywords:** germline testing, incidental finding, multiple primary malignancies, papillary thyroid carcinoma, testicular seminoma

## Abstract

The coexistence of testicular seminoma and papillary thyroid carcinoma (PTC) is uncommon. We report a 32-year-old man with localized pure testicular seminoma treated by right orchiectomy. Staging computed tomography, performed before adjuvant carboplatin, incidentally demonstrated bilateral thyroid lesions. Subsequent evaluation revealed a highly suspicious right thyroid nodule and suspicious cervical lymph nodes. Surgery demonstrated bilateral multifocal PTC with lymphatic invasion and cervical lymph node metastases. Radioiodine therapy was subsequently administered. Comprehensive germline testing using a 90-gene cancer-predisposition panel identified no pathogenic or likely pathogenic variants. The detection of thyroid abnormalities before systemic seminoma treatment makes a treatment-related origin of the PTC highly unlikely. This case highlights the importance of evaluating clinically relevant incidental findings during oncological staging and illustrates the unresolved relationship between testicular germ-cell tumors (TGCTs) and thyroid malignancies.

## Introduction

Testicular germ-cell tumors (TGCTs) are the most common solid malignancies affecting young adult men, with seminoma representing one of the predominant histological subtypes. Testicular cancer is characterized by excellent long-term survival, particularly in patients with localized disease. Consequently, treatment-related morbidity and the occurrence of second primary malignancies have become increasingly relevant aspects of survivorship care [1,2].

Papillary thyroid carcinoma (PTC) represents the predominant histological subtype of differentiated thyroid carcinoma and likewise generally carries a favorable prognosis, particularly in younger patients [3,4]. Although seminoma and PTC are individually encountered in young adults, their occurrence in the same patient appears to be uncommon.

Population-based studies have demonstrated an increased incidence of second solid malignancies among testicular cancer survivors [5-8]. Thyroid cancer has repeatedly emerged as one of the sites with an increased relative incidence. In a Norwegian population-based cohort of 5,625 testicular cancer survivors, thyroid cancer remained significantly increased even among patients treated with surgery alone, with a standardized incidence ratio of 4.95 (95% CI, 1.86-13.18), suggesting that therapeutic exposure alone may not fully account for the observed association [8].

The available case-based literature is limited and heterogeneous. Previous reports have described PTC in patients with pure seminoma, nonseminomatous germ-cell tumors, and complex sequences of multiple testicular germ-cell tumors [9-12]. Proposed explanations include treatment-related carcinogenesis, increased surveillance and incidental detection, shared genetic susceptibility, and coincidental occurrence.

We report the case of a young man with localized pure testicular seminoma in whom bilateral thyroid abnormalities were incidentally identified during initial oncological staging, before adjuvant systemic therapy, ultimately leading to the diagnosis of bilateral multifocal, lymph-node-positive PTC. Comprehensive germline testing did not identify a recognized monogenic cancer-predisposition syndrome. The close temporal relationship between the two malignancies provides an opportunity to examine potential mechanisms underlying this unusual association.

## Case presentation

A 32-year-old man underwent urological evaluation of a persistent sonographically indeterminate lesion of the right testis. Serial ultrasonographic examinations between July and October 2025 demonstrated a persistent focal abnormality. Because malignancy could not be reliably excluded, surgical exploration was recommended. The family history was unremarkable for malignancy.

Serum germ-cell tumor markers were within the reference ranges at initial assessment (Table 1). 

**Table 1:** Serum tumor marker levels at initial assessment and postoperative follow-up

Time point	Laboratory parameter	Result	Reference range
Initial assessment	Alpha-fetoprotein	2.3 ng/ml	<7 ng/mL
Initial assessment	Beta-human chorionic gonadotropin	<0.2 IU/L	<2.6 IU/L
Initial assessment	Lactate dehydrogenase	204 U/L	<248 U/L
Postoperative follow-up	Alpha-fetoprotein	1.4 ng/ml	<7 ng/ml
Postoperative follow-up	Beta-human chorionic gonadotropin	<0.2 IU/L	<2.6 IU/L
Postoperative follow-up	Lactate dehydrogenase	209 U/L	<248 U/L

In October 2025, the patient underwent right inguinal testicular exploration with intraoperative frozen-section examination. Frozen-section analysis demonstrated seminoma, and right orchiectomy was subsequently performed. A contralateral left testicular biopsy was also obtained.

Definitive histopathological examination demonstrated a 3.5-cm pure seminoma of the right testis with involvement of the rete testis and associated germ cell neoplasia in situ. No vascular invasion was identified, the tunica albuginea was not infiltrated, and resection margins were negative. No nonseminomatous germ-cell tumor component was identified. The tumor was classified as pT1 pNx L0 V0 R0; serum tumor markers corresponded to S0. Histological examination of the contralateral testicular biopsy demonstrated tumor-free testicular tissue with preserved spermatogenesis and no evidence of germ cell neoplasia in situ.

Postoperative serum tumor markers remained within the reference ranges (Table 1). 

Postoperative staging was performed in November 2025 and included abdominal MRI and contrast-enhanced CT of the chest. Neither examination demonstrated metastatic seminoma. Although the neck was not completely included in the thoracic CT acquisition, the imaged thyroid demonstrated an incidental hypodense lesion measuring approximately 15 mm in the right thyroid lobe (Figure 1) and an additional approximately 8-mm hypodense lesion in the left lobe. Dedicated thyroid ultrasonography was recommended. The thyroid abnormalities were therefore incidental imaging findings during oncological staging.

**Figure 1:**
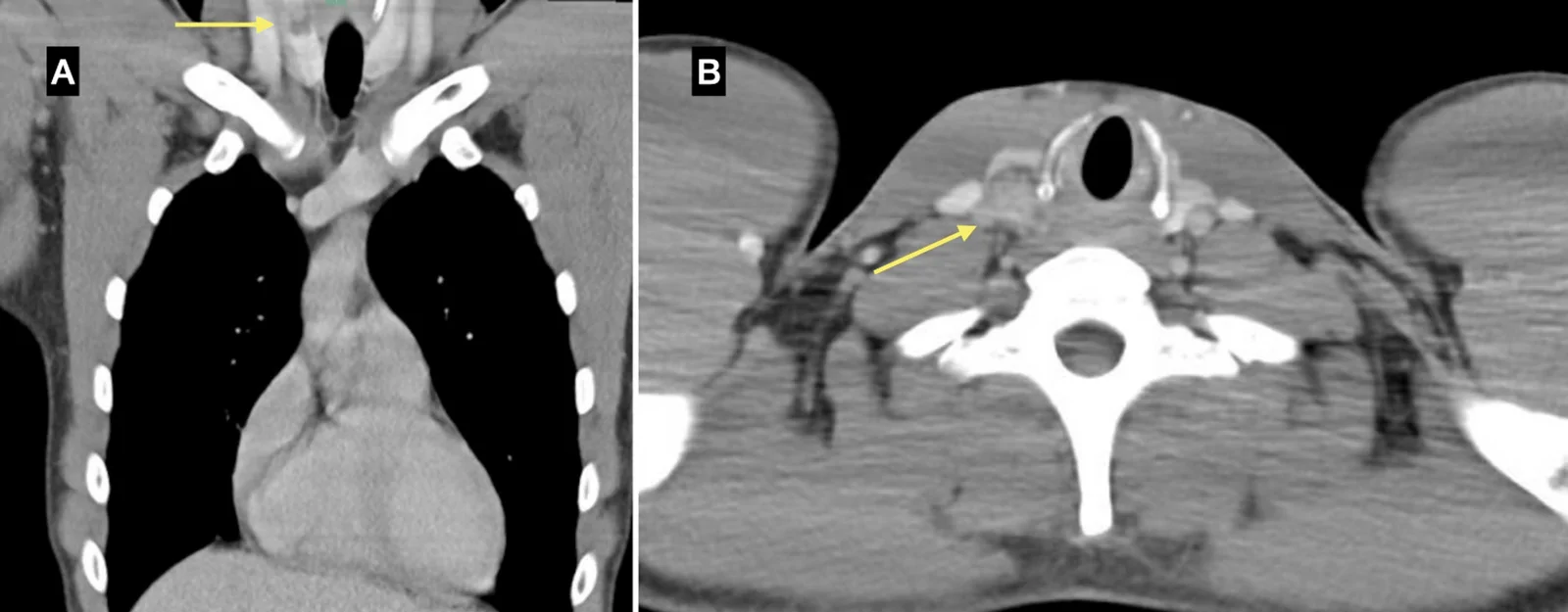
Incidental thyroid abnormality detected on staging CT. (A) Coronal and (B) axial contrast-enhanced CT images obtained during staging for testicular seminoma demonstrate the incidentally detected right thyroid lesion (arrows). The thyroid gland was only partially included in the scan range.

The patient subsequently received a single adjuvant administration of carboplatin in December 2025.

Dedicated thyroid evaluation was performed in January 2026. The patient was clinically euthyroid and reported no thyroid-specific symptoms. Preoperative thyroid-related laboratory parameters, including thyroid-stimulating hormone, thyroglobulin, and calcitonin, were within the respective reference ranges. Ultrasonography demonstrated a lobulated, hypoechoic, predominantly solid nodule with macrocalcifications and a peripheral fluid component in the upper right thyroid lobe, classified as highly suspicious (Thyroid Imaging Reporting and Data System (TI-RADS) 5) (Figure 2A). Thyroid scintigraphy demonstrated reduced tracer uptake corresponding to the right-sided lesion. A small cystic lesion was additionally identified in the left thyroid lobe.

**Figure 2:**
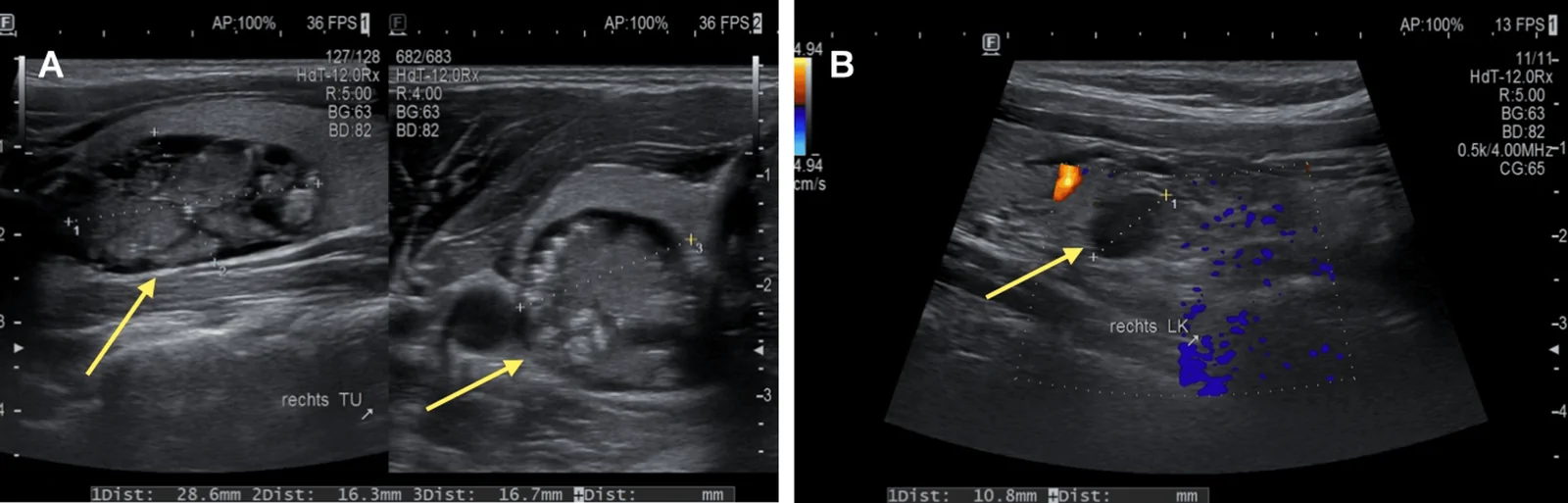
Ultrasonographic findings of the right thyroid lesion and suspicious cervical lymphadenopathy. (A) Ultrasonography demonstrates a predominantly solid, heterogeneous right thyroid nodule with cystic components and calcifications, shown in longitudinal and transverse planes. (B) Color Doppler ultrasonography demonstrates a suspicious right lateral cervical lymph node. Yellow arrows indicate the respective lesions.

Subsequent surgical ultrasonographic assessment demonstrated suspicious right lateral cervical lymph nodes, particularly in level III, including a morphologically abnormal lymph node demonstrated on Doppler imaging (Figure 2B). Preoperative fine-needle aspiration cytology was not pursued because, following surgical assessment, cytological confirmation was not expected to alter the planned operative strategy.

In March 2026, the patient initially underwent right hemithyroidectomy with intraoperative frozen-section examination. Frozen-section analysis confirmed PTC, prompting completion thyroidectomy together with central and right lateral cervical lymph-node dissection.

Definitive histopathological examination demonstrated bilateral multifocal PTC. The dominant right-sided carcinoma measured 27 mm and showed characteristic papillary architecture, nuclear features of PTC, psammomatous calcifications, and lymphatic invasion. An additional 8-mm PTC was identified in the left thyroid lobe. Five of nine examined lymph nodes contained metastatic PTC, including right lateral cervical nodal involvement. No vascular or perineural invasion was identified, and resection margins were negative. The final pathological classification was pT2(m) pN1b (5/9) L1 V0 Pn0 R0.

The postoperative course was uncomplicated. Bilateral vocal-cord mobility was preserved, and postoperative calcium and parathyroid hormone concentrations remained within the reference range. Following thyroid surgery, adjuvant radioiodine therapy was administered after stimulation with recombinant human thyroid-stimulating hormone and was well tolerated. Post-therapy imaging demonstrated cervical iodine uptake, and continued follow-up was arranged.

In June 2026, because of the unusual occurrence of two distinct primary malignancies at a young age, the patient underwent comprehensive germline genetic testing using a 90-gene panel for hereditary predisposition to adult solid tumors. The panel included CHEK2 and BRAF among other cancer-predisposition genes. No pathogenic or likely pathogenic germline variants (class 4/5) were identified. No clinically relevant deletions or duplications were detected, and no variants of uncertain significance (class 3) were reported. No separate somatic tumor molecular testing was performed. Thus, the genetic evaluation did not identify a molecular genetic cause or a recognizable monogenic hereditary cancer predisposition accounting for the coexistence of seminoma and papillary thyroid carcinoma.

The clinical course and key diagnostic and therapeutic events are summarized in Table 2.

**Table 2:** Clinical timeline Abbreviations: TI-RADS, Thyroid Imaging Reporting and Data System; PTC, papillary thyroid carcinoma.

Time point	Clinical event
Initial assessment	Persistent indeterminate right testicular lesion on ultrasonography
Seminoma diagnosis and primary treatment	Right orchiectomy: pure seminoma, 3.5 cm, pT1 pNx L0 V0 R0, S0
Initial staging	Staging MRI/CT: no seminoma metastases; incidental bilateral thyroid lesions
Adjuvant seminoma treatment	Adjuvant carboplatin
Thyroid assessment	Thyroid ultrasound: TI-RADS 5 right nodule and suspicious cervical lymph nodes
Thyroid treatment	Thyroidectomy and cervical lymph-node dissection: bilateral multifocal PTC, pT2(m) pN1b (5/9) L1 V0 Pn0 R0
Adjuvant thyroid treatment	Adjuvant radioiodine therapy
Genetic evaluation	90-gene germline panel: no pathogenic/likely pathogenic variants

## Discussion

The present case describes the near-synchronous occurrence of localized pure testicular seminoma and bilateral multifocal, lymph-node-positive PTC in a young man. Notably, the thyroid abnormality was incidentally detected during initial seminoma staging, before systemic treatment. The coexistence of TGCT and PTC has previously been reported, although the case-level literature remains limited and heterogeneous. Three patients with germ-cell tumors and thyroid malignancies have been described, predominantly in the context of nonseminomatous disease, with one patient having pure seminoma [9]. Two unrelated patients were subsequently reported with an unusual sequence of testicular teratoma, contralateral seminoma, and later PTC [10]. Three metachronous thyroid malignancies following testicular cancer have also been reported [11]. More recently, PTC was detected during restaging after chemotherapy for metastatic mixed nonseminomatous germ-cell cancer, with BRAF V600E identified in both tumors [12].

Testicular cancer survivors have an established long-term excess risk of subsequent solid malignancies [5-7]. However, the temporal sequence in the present patient makes a treatment-related origin of the PTC highly unlikely. The thyroid abnormalities were already visible on staging CT performed only 12 days after orchiectomy and approximately four weeks before the administration of adjuvant carboplatin. The patient had not received radiotherapy. Treatment-related solid malignancies generally occur after latency periods measured in years or decades rather than days or months [6,7]. Treatment exposure may therefore not fully explain the observed epidemiological relationship between testicular and thyroid malignancies. A significantly increased incidence of thyroid cancer has been reported even among testicular cancer survivors managed with surgery alone, with a standardized incidence ratio of 4.95 (95% CI, 1.86-13.18) [8]. Earlier population-based analyses similarly demonstrated elevated relative incidences of thyroid cancer following testicular cancer [5,13]. These estimates should nevertheless be interpreted cautiously. Thyroid cancer represents a relatively uncommon outcome in these cohorts, resulting in small event numbers and wide confidence intervals. Therefore, the magnitude and clinical significance of the observed association remain uncertain.

Increased diagnostic surveillance may contribute to the observed association between testicular and thyroid cancer, as staging or follow-up imaging can reveal otherwise occult thyroid lesions. In the present patient, thyroid abnormalities were incidentally detected during seminoma staging. However, definitive pathology demonstrated clinically significant disease, with a 27-mm dominant PTC, bilateral multifocal disease, lymphatic invasion, and cervical lymph-node metastases (5/9), requiring thyroidectomy, lymph-node dissection, and radioiodine therapy.

No established monogenic cancer-predisposition syndrome is currently recognized as characteristically predisposing to both TGCT and PTC. Inherited pathogenic variants in CHEK2 have been associated with susceptibility to TGCT, although this observation does not establish a common hereditary mechanism linking TGCT and PTC [14]. In the present patient, a comprehensive 90-gene germline cancer-predisposition panel revealed no pathogenic or likely pathogenic variants, clinically relevant copy-number alterations, or variants of uncertain significance. This result reduces the likelihood of a currently recognizable monogenic hereditary cancer-predisposition syndrome but does not exclude more complex or currently unidentified genetic susceptibility. Accordingly, the genetic findings should be interpreted as showing no identifiable monogenic hereditary cancer predisposition rather than excluding a potential genetic relationship between the two malignancies.

Current testicular-cancer follow-up strategies do not include routine thyroid ultrasonography. However, this case highlights the importance of appropriately evaluating incidental thyroid abnormalities detected during oncological staging. Given the potential contribution of surveillance-related detection and overdiagnosis, these observations should be regarded as hypothesis-generating. Further studies are needed to clarify the nature and clinical significance of the reported association between TGCT and thyroid malignancies.

## Conclusions

The near-synchronous occurrence of localized pure testicular seminoma and bilateral multifocal, node-positive PTC represents an uncommon presentation of two distinct primary malignancies in a young adult. The thyroid abnormalities were detected during initial staging before adjuvant systemic therapy, making a treatment-related origin of the PTC highly unlikely. Comprehensive germline testing identified no recognizable monogenic cancer-predisposition syndrome. This case highlights the clinical importance of appropriately evaluating incidental thyroid abnormalities detected during oncological staging. The observed coexistence of TGCT and PTC should be regarded as hypothesis-generating, and further studies are needed to clarify whether the reported association reflects shared susceptibility, surveillance-related detection, or coincidental occurrence.
